# The Impact of Preoperative Weight Loss on Long-term Success: 5-year Outcomes After Metabolic Bariatric Surgery

**DOI:** 10.1007/s11695-025-08476-2

**Published:** 2026-01-06

**Authors:** Kayleigh A. M. van Dam, Cathelijne Kam, Marijn T. F. Jense, Geert H. J. M. Verkoulen, Pieter P. H. L. Broos, Evelien de Witte, Jan Willem M. Greve, Evert-Jan G. Boerma

**Affiliations:** 1 Zuyderland Medical Center, Heerlen, Netherlands; 2 NUTRIM, Maastricht University, Maastricht, Netherlands; 3 Nederlandse Obesitas Kliniek, Heerlen, Netherlands; 4 NUTRIM, Maastricht University Medical Center, Maastricht, Netherlands

## Abstract

**Introduction:**

Preoperative weight loss has been suggested as a predictor of postoperative weight loss outcomes after Metabolic Bariatric Surgery (MBS). While previous studies focused on outcomes up to 3 years, longer-term results remain limited. This study evaluates the association between preoperative weight loss and total weight loss (%TWL) up to five years postoperatively.

**Methods:**

This single-center retrospective study included 765 patients who underwent primary MBS between June 2017 and august 2019. Patients were stratified into quartiles based on preoperative weight loss. Follow-up data on %TWL were analyzed at 3 and 6 months and 1 through 5 years postoperatively.

**Results:**

The median age was 45 (35-52) and the majority were female (76.6%). Most patients underwent ring augmented Roux-en-Y Gastric Bypass (91.1%). Follow-up was 99.6% at 1 year and 65.9% at 5 years. Quartiles were defined as Q1: <4.4%, Q2:4.4-6.2%, Q3: 6.2-8.2% and Q4: > 8.2% preoperative weight loss. After 5 years %TWL was 32.4 (Q1), 32 (Q2), 32 (Q3) and 31.6 (Q4).

**Conclusion:**

Greater preoperative weight loss was associated with significant higher %TWL up to two years (quartiles) and four years (low vs high) after MBS, but no significant differences were found after five years of follow up. The impact of preoperative weight loss on long-term outcomes was consistent across subgroups. Female sex, RYGB procedure and younger age were general predictors for greater %TWL.

**Supplementary Information:**

The online version contains supplementary material available at 10.1007/s11695-025-08476-2.

## Introduction

Metabolic Bariatric Surgery (MBS) remains the most effective treatment in patients with obesity, leading to substantial and sustained weight loss [1, 2]. Achieving and preserving this weight loss after MBS improves overall quality of life and reduces associated obesity related medical problems, such as type 2 diabetes, hypertension and dyslipidemia [1–4]. The most commonly performed bariatric procedures worldwide are the Roux-en-Y Gastric Bypass (RYGB) and the Sleeve Gastrectomy (SG) [2, 5]. Although both procedures are effective, long-term data suggest that RYGB is associated with a greater percentage total weight loss (%TWL) compared to SG [2, 6].

Optimizing patient outcomes remains an important aspect of bariatric care. The role of preoperative weight loss has been the subject of debate, as it might enhance postoperative outcomes [3, 4, 7–11]. Preoperative weight loss is thought to reduce perioperative risks, among others by decreasing liver size and visceral fat [3, 8, 9]. Another hypothesis focuses on behavioral aspects, namely that patients who achieve more weight loss preoperatively may be more motivated and might be more successful in sustaining weight loss [3, 7]. Furthermore, identifying which patient subgroups benefit most from preoperative weight loss could help tailor preoperative strategies.

The available literature has been inconsistent regarding the relationship between preoperative weight loss and postoperative outcomes. Some studies found that patients who lost weight prior to surgery were more likely to achieve more weight loss while other studies observed no significant differences at all [3, 7–9]. The studies are further limited due to inconsistent definitions of preoperative weight loss and the short follow-up durations up to three years [3, 4, 7–11].

The aim of the present study was to evaluate the association between preoperative weight loss and total weight loss up to five years after MBS. In addition, we aimed to identify patient characteristics associated with successful long-term weight loss outcomes.

## Methods

### Patient Selection

This is a retrospective study including all consecutive patients that underwent primary MBS (ring augmented RYGB or SG) between June 2017 and August 2019 in Hospital X. Patients were excluded if they underwent other primary bariatric procedures such as adjustable gastric banding (AGB) or one-anastomosis gastric bypass (OAGB) or if they underwent revisional or conversional surgery. In addition, patients that were converted from SG to raRYGB during the 5-year follow-up period were also excluded.

All patients were preoperatively screened and approved by a multidisciplinary team. Patients followed comprehensive interdisciplinary program before and after surgery at the Dutch Obesity Clinic. The Dutch Obesity Clinic has the following preoperative weight loss policy: patients were requested to lose at least 3.5 kg and in patients with a BMI of 55 or higher the required minimal weight loss was 10 kg. The one-year results of this patient series have already been published [3].

## Definitions

For all weight-related variables and outcomes, definitions were predefined to ensure consistency.

Baseline weight was defined as the highest weight recorded between screening and session 1 of the preoperative program, representing the highest weight prior to the start of the treatment. Surgery weight was measured on the day of the bariatric procedure.

Preoperative weight loss (%preopWL) measured the weight loss before surgery using baseline weight and surgery weight. It was calculated as:

$$\:\%preopWL=\left(\frac{baseline\:weight-surgery\:weight}{baseline\:weight}\right)*100$$

Total weight loss (%TWL) measured the overall weight loss from baseline to follow-up. Each follow-up time point (e.g. 1, 3 or 5 years) used the patient’s weight at that specific time to calculate %TWL. It was calculated as:

$$\:\%TWL=\left(\frac{baseline\:weight-follow-up\:weight}{baseline\:weight}\right)*100$$

Postoperative weight loss measured the weight loss after surgery without taking into account the preoperative weight loss. Each follow-up time point used the patient’s weight at that specific time compared to the weight at the moment of surgery. It was calculated as:

$$\:postoperative\:\%WL=\left(\frac{surgery\:weight-follow-up\:weight}{surgery}\right)*100$$

To evaluate the impact of preoperative weight loss on outcomes, patients were stratified into four groups (Q1, Q2, Q3, and Q4) based on quartiles of %preopWL, ranging from lowest (Q1) to highest (Q4) preoperative weight loss. Additionally, a secondary stratification was applied, grouping patients into two broader categories: low preoperative weight loss (Q1 + Q2) and high preoperative weight loss (Q3 + Q4).

## Data Collection

All patient data were retrospectively collected from electronic patient files at hospital X. The baseline data included age, gender, height, weight, BMI, and obesity associated medical problems. The obesity associated medical problems included hypertension, diabetes mellitus, obstructive sleep apnea syndrome (OSAS), dyslipidemia and arthrosis defined according to the standardized outcomes in MBS [12].

The primary outcome measure of %TWL after 5-year follow-up was calculated using the weight after 5 years of follow-up compared to the baseline weight. The secondary outcomes consisted of preoperative weight loss (%preopWL), %TWL at 1, 2, 3 and 4 years and early (< 30 days) and late (*≥* 1 year) complications. Complications were classified according to the Clavien-Dindo (CD) classification [13].

### Statistical Analysis

Statistical analysis was performed using IBM SPSS Statistics for Windows, version 29.0. Categorical variables were presented as frequencies with percentages. Continuous variables were presented as mean *±* standard deviation (SD) for normal distributed variables and median and interquartile range (IQR) for a skewed distribution. Differences between subgroups were tested using Chi-square or Fisher’s exact test for categorical variables, and independent t-test or Mann-Whitney U test for continuous variables.

The Kruskal-Wallis test was used to assess whether the %TWL differed significantly across quartiles at each follow-up time point between the four groups (Q1-Q4). These year-by-year comparisons were at multiple time points and thus a Bonferroni correction was applied, resulting in an adjusted significance threshold of *p* < 0.007. Post-hoc comparisons were performed for significant timepoints. The Mann-Whitney U test was used to assess whether the %TWL differed between two groups. To assess the association between preoperative weight loss and %TWL over time, accounting for repeated measures within patients, a linear mixed model (LMM) was used. Time was included as a repeated factor and fixed and random effects were used. Interaction terms were tested to evaluate whether the effect of preoperative weight loss varied by subgroups characteristics. The LMM included time, preoperative WL group, and baseline covariates (age, gender, BMI, procedure type) as fixed effects.

A multivariable linear regression analysis was performed to assess whether preoperative weight loss was associated with %TWL at 1- and 5-years follow-up after adjustment for age, gender, BMI and procedure type. A *p*-value of < 0.05 was considered statistically significant. Missing data were reported as such. The LMM accounts for missing follow-up data, as it handles incomplete observations under the assumption of missing at random.

## Results

A total of 765 patients were analyzed of whom 586 were female (76.6%) and 179 male (23.4%). The preoperative demographical data are summarized in Table 1. The cohort’s median age was 45 years (35–52) and median baseline BMI was 42.1 kg/m^2^ (39.6–46). Regarding the obesity associated medical problems, hypertension was present in 230 patients (30.1%), diabetes mellitus in 23 (3%), OSAS in 69 (9%), dyslipidemia in 34 (4.4%) and arthrosis in 38 patients (5%). The majority underwent raRYGB (91.1%) and 68 patients underwent SG (8.9%). At the day of the procedure the median BMI was 39.4 (36.9–42.8).

**Table 1:** Baseline characteristics total study population

	N = 765
Age (years)	45 (35 – 52)
Gender	
* Male*	179 (23.4)
* Female*	586 (76.6)
Height (cm)	169 (164 – 174)
Baseline weight (kg)	121.8 (110.8 – 136.8)
Baseline BMI (kg/m^2^)	42.1 (39.6 - 46)
Weight at surgery (kg)	114 (103 – 127)
BMI at surgery (kg/m^2^)	39.4 (36.9 - 42.8)
%preopWL*	6.2 (-2.71 – 24.31)
Type of surgery	
* raRYGB*	697 (91.1)
* Sleeve Gastrectomy*	68 (8.9)
Obesity associated medical problems	
* Hypertension*	230 (30.1)
* Diabetes mellitus*	23 (3)
* OSAS*	69 (9)
* Dyslipidemia*	34 (4.4)
* Arthrosis*	38 (5)

Data are presented as median (IQR) or N (%)

*Instead of IQR the full range with minimum and maximum value is used for %preopWL

### Pre-operative Weight Loss

Median %preopWL was 6.2 (full range − 2.71–24.31). The patients were divided into four groups, based on the four quartiles for %preopWL, resulting in Q1: <4.4%, Q2: 4.4–6.2%, Q3: 6.2–8.2%, and Q4: > 8.2%. As shown in supplementary Table 1 the baseline characteristics are comparable among the four quartiles except for age. Patients in the highest preoperative weight loss quartiles were on average older than those in the lower quartiles.

## Follow-up and Weight Loss Results

All patients had the opportunity for 5-year follow-up. Follow-up completion rates remained high in the first year (97.3%) but gradually declined to 64.9% at five years, as shown in Table 2. The median %TWL for all patients at five-year follow-up was 32%.

**Table 2:** Follow-up and %TWL during 5-year follow-up

	Follow-up	%TWL
3 month follow-up	762/765 (99.6)	21.8 (19.2–24.6)
6 month follow-up	752/765 (98.3)	30.6 (27.1–34.5)
1 year follow-up	744/765 (97.3)	36.7 (32.1–40.7)
2 years follow-up	639/764 (83.6)	36.8 (31.1–42)
3 years follow-up	598/763 (78.4)	34.8 (28.6–40.9)
4 years follow-up	549/761 (72.1)	33.4 (27.1–39.4)
5 years follow-up	493/760 (64.9)	32 (26.2–38.5)

**Fig. 1:**
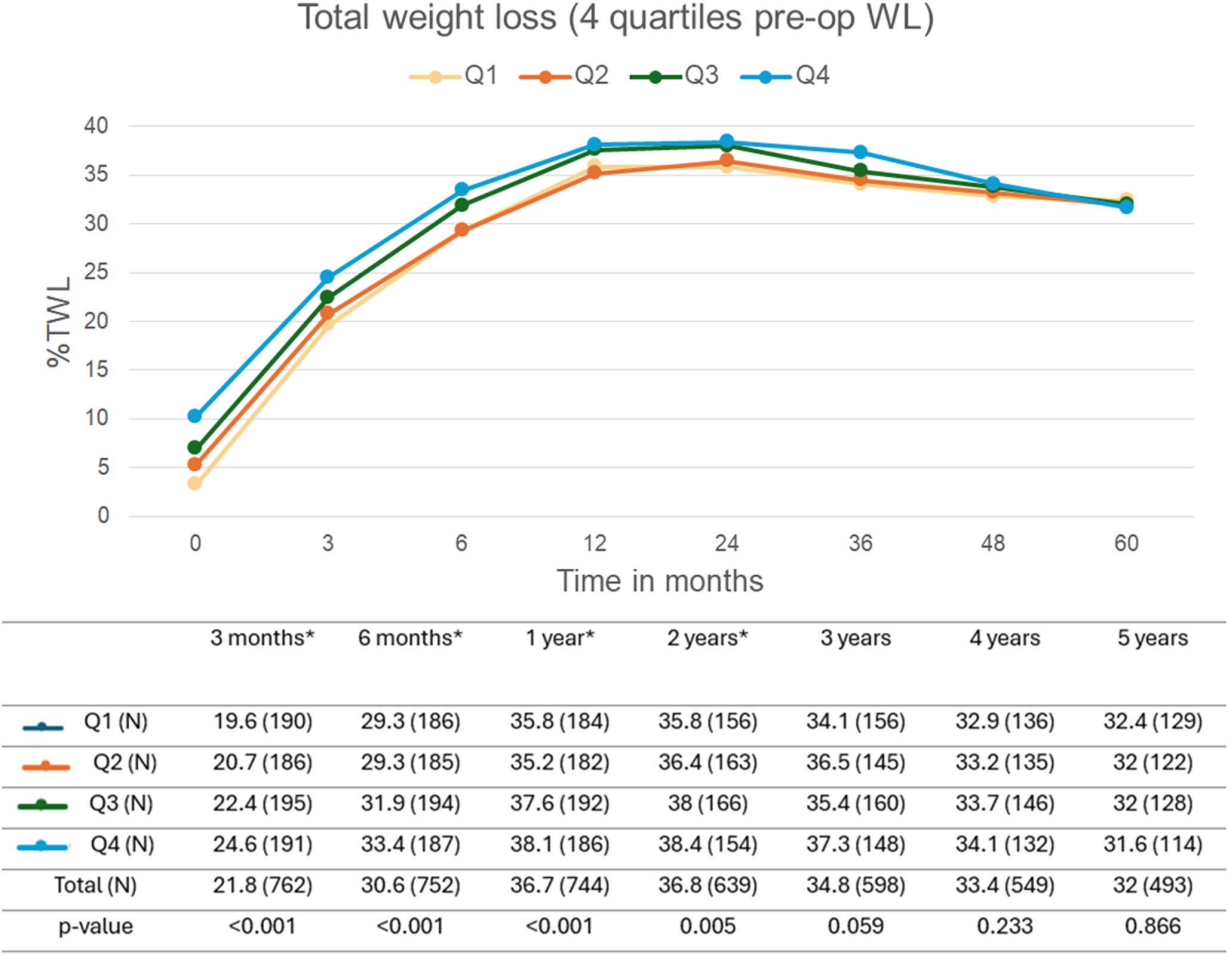
Total weight loss (%TWL) during follow-up, based on the four pre-operative weight loss quartiles. Data is presented as median (n), the n shows the follow-up rate and distribution. P-value calculated with Kruskal-Wallis test. *Significant result with a *p*-value of < 0.005

While all preoperative weight loss quartiles followed a similar postoperative weight loss trajectory, significant differences in %TWL were observed up to two years postoperatively even after Bonferroni correction, as shown in Fig. 1. At 1 year, patients in Q4 achieved a median %TWL of 38.1 compared to 35.8% in Q1 (Δ 2.3%). At 5 years, patients in Q4 achieved a median %TWL of 31.6 compared to 32.4% in Q1 (Δ -0.8%). The patients in the higher preoperative weight loss quartiles achieved greater %TWL compared to those in the lowest quartiles. Differences diminished after two years, with no significant differences at three-, four- and five-year follow-up.

**Fig. 2:**
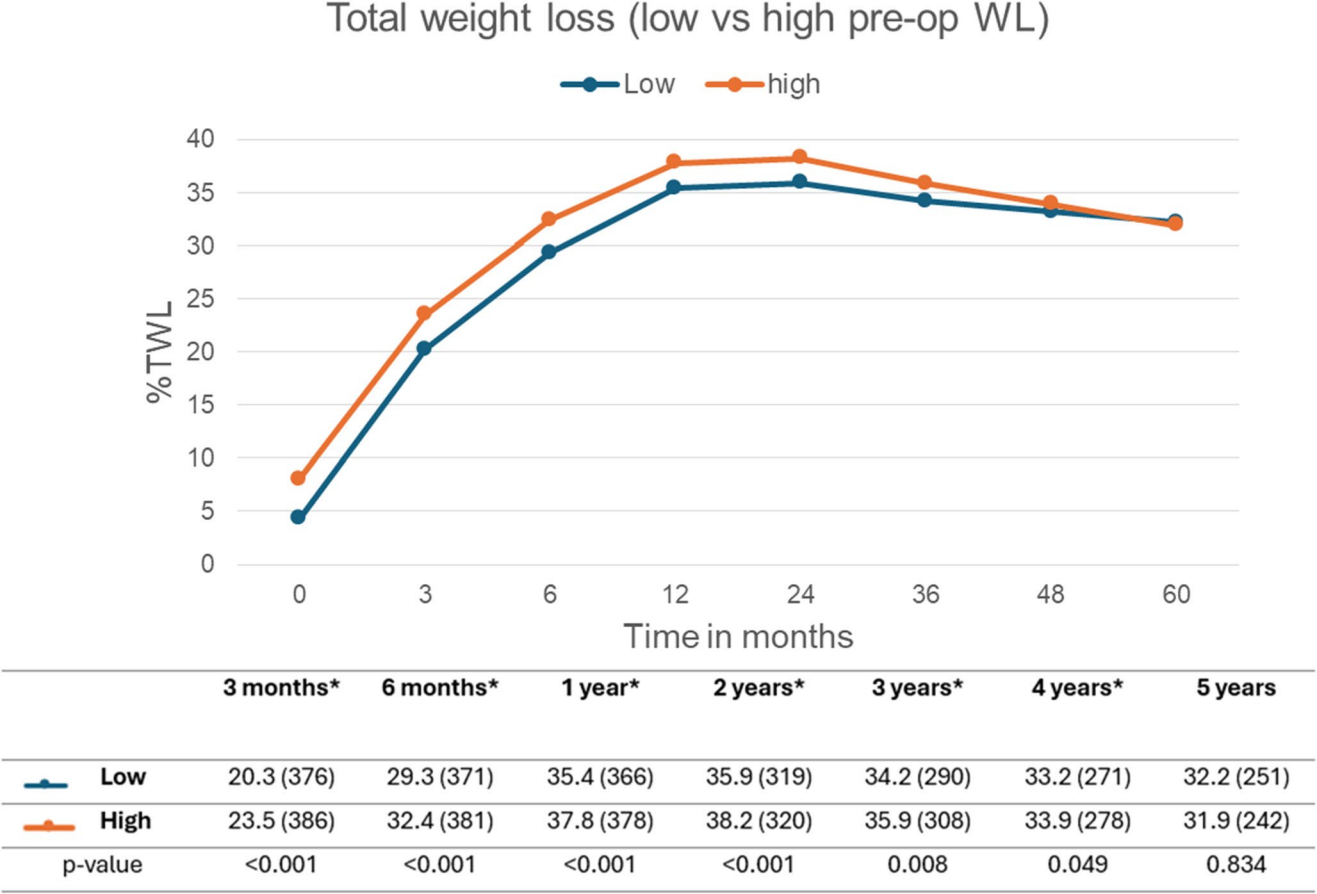
Total weight loss (%TWL) during follow-up, based on high vs. low preoperative weight loss. Data is presented as median (n), the n shows the follow-up rate and distribution. *P*-value calculated with Mann-Whitney U Test. *Significant result with a *p*-value of < 0.005

To further assess the effect of preoperative weight loss patients were also divided into two categories: low and high preoperative weight loss, with low comprising patients from Q1 and Q2, and high containing patients from Q3 and Q4. As shown in Fig. 2, the high group achieved a significantly higher %TWL through the first four postoperative years. By year five, the %TWL showed no significant differences between the two groups with a TWL of 32.2% in the high group vs. 31.9% in the low group (*p* = 0.834).

**Fig. 3:**
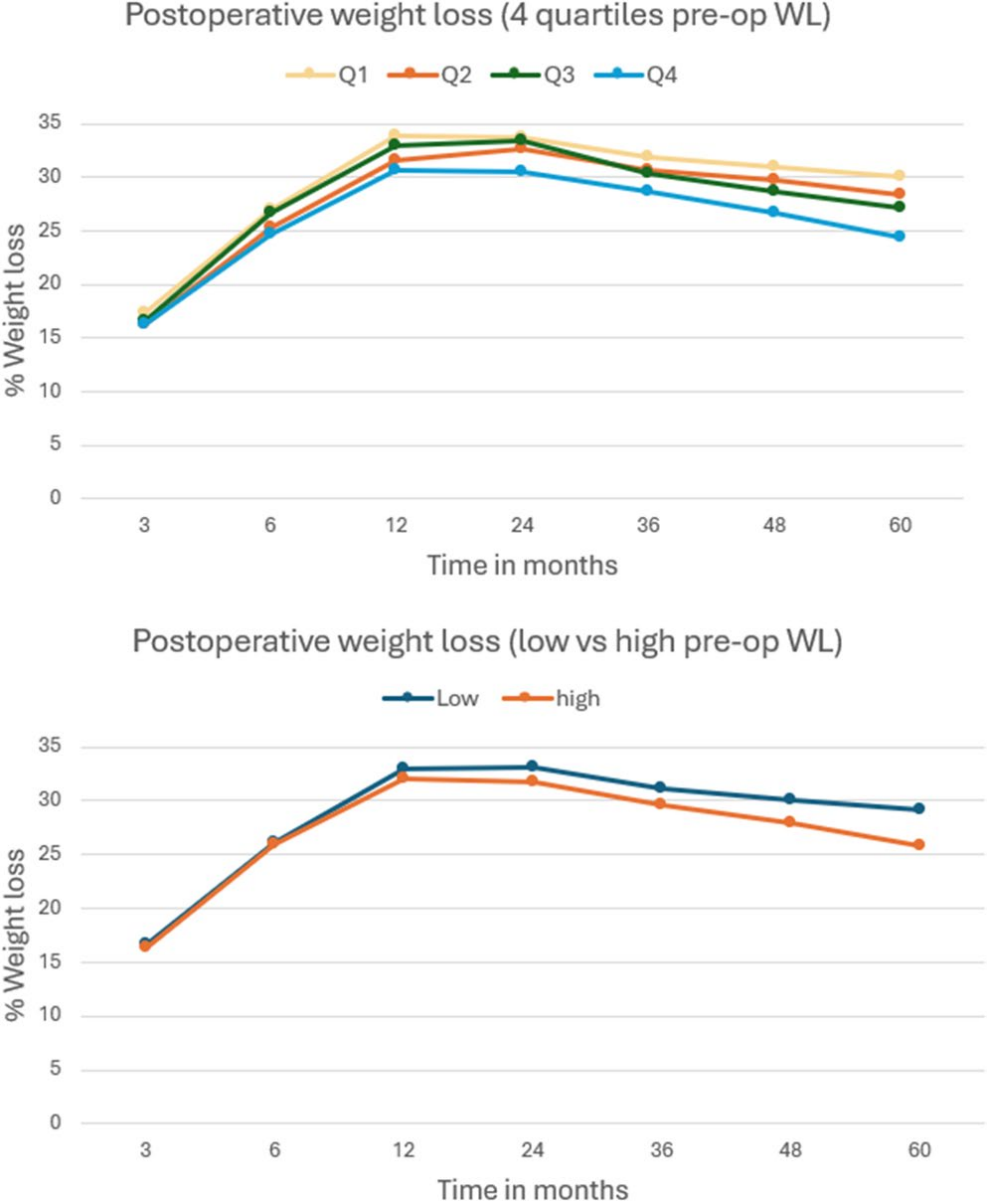
Postoperative weight loss (%WL) during follow-up. % Weigt loss calculated from surgery weight, excluding the preoperative weight loss component. Note that patients with greater preoperative weight loss tended to lose less after surgery, reversing the rank order observed in total %TWL

Regarding %postoperative WL, without the pre-operative component, the %WL after five years was 30.1 in Q1, 28.3 in Q2, 27.1 in Q3 and 24.4 in Q4. The low vs. high pre-operative WL division showed a WL of 29.2 and 25.8% after five years. The different preoperative weight loss groups, both quartiles and dichotomous, followed an overall similar postoperative weight loss curve (Fig. 3).

## Factors Associated with Weight Loss

In the final linear mixed model, several interaction effects were tested to determine whether the impact of preoperative weight loss on %TWL over time varied by patient characteristics. No significant interactions were found between the four quartiles and gender (*p* = 0.146), procedure type (*p* = 0.977), age (*p* = 0.335) and BMI (*p* = 0.476) in predicting %TWL over time as shown in Table 3. Assessing age and BMI based on low vs. high instead of continuous values also showed no significant interactions (*p* = 0.382 and *p* = 0.338). This indicates that the effect of preoperative weight loss on %TWL was consistent across these subgroups and over time.

**Table 3:** Factors associated with greater weight loss, stratified by preoperative weight loss results

	4 quartiles of preopWL		Low vs high preopWL	
	F	*P*-value	F	*P*-value
Gender	1.798	0.146	2.685	0.101
Age				
* Continuous*	1.132	0.335	1.203	0.273
* <45 vs > 45*	1.022	0.382	1.189	0.276
Type of procedure	0.067	0.977	0.169	0.681
BMI during screening				
* Continuous*	0.833	0.476	0.306	0.580
* <41.6 vs > 41.6*	1.125	0.338	0.281	0.596

F statistics from linear mixed model, P-value calculated with linear mixed model. Gender: male = 0, female = 1, Type of procedure: RYGB = 0, Sleeve Gastrectomy = 1

When patients were grouped into two broader categories, namely low vs. high preoperative weight loss the interaction effects were also not significant (Table 3). No statistically significant interaction with gender was found (*p* = 0.057), though males in the high WL group had a nominally higher %TWL than females with a 3.2% difference.

Independent of the quartiles or low vs. high WL groups, several baseline characteristics were significantly associated with %TWL over time (Table 4). Being female was a strong predictor of greater %TWL (*p* < 0.001). In addition, younger age (*p* < 0.001) was significant when using continuous age as each year of age is associated with 0.069% lower TWL. The RYGB procedure was a significant predictor for weight loss as it achieved 2.1% more TWL as the sleeve procedure (*p* < 0.001). The other variables of continuous and binary BMI were not significantly associated with higher weight loss over time. Multivariable regression confirmed that preoperative weight loss was an independent predictor of %TWL at 1 year (β = 0.45, *p* < 0.001) but not at 5 years (β= -0.46, *p* = 0.415) (supplementary Table 2). Age remained a negative predictor at both timepoints, while gender and procedure type were significantly at 1 year. Baseline BMI was not significant in either model.

**Table 4:** General predictors for greater weight loss

	F	Estimate (β)	*P*-value
Gender (female)	16.393	0.947	<0.001
Age			
* Continuous*	21.333	-0.069	<0.001
* Low age*	2.833	0.582	0.092
Type of procedure (RYGB)	37.731	2.093	<0.001
BMI during screening			
* Continuous*	1.577	-0.030	0.209
* Low BMI*	3.271	0.492	0.071

F statistics from linear mixed model, estimate is the effect on %TWL. For instance gender (female) indicates that female patients have 0.947% more %TWL than male patients.

*P*-value calculated with linear mixed model. Low age: <45 years, low BMI: <41.6 kg/m^2^

### Complications

A total of 108 patients experienced short- and long-term complications (Table 5). Among these, 22 complications (2.9%) occurred within the first 30 days postoperatively. Most of the complications, 93 (10.8%), were long-term.

**Table 5:** Short- and long-term complications

Variables	N = 765
Patients with complications	108 (14.1)
Short-term complications	
Patients with short-term complications	22 (2.9)
Short-term (≤ 30 days) complications according to Clavien-Dindo	
* 1*	1 (0.1)
* 2*	4 (0.5)
* 3a*	5 (0.7)
* 3b*	12 (1.6)
Long-term complications	
Patients with long-term complications	86 (11.2)
Long-term (>30 days) complications according to Clavien-Dindo	
* 2*	1 (0.1)
* 3a*	12 (1.6)
* 3b*	80 (10.5)

Of the short-term complications, 10 were classified as *≤* CD3a and 12 were classified as *≥* CD3b. The short-term complications consisted of anastomotic leakage (*n* = 7), intra-abdominal abscesses (*n* = 2), perforation (*n* = 2), hematoma (*n* = 3), postoperative bleeding (*n* = 4), omental infarction (*n* = 1), pneumonia (*n* = 1) and food impaction (*n* = 2).

There were 86 patients (11.2%) with 93 long-term complications of which 80 were *≥* CD3b. The most frequent complication was internal herniation (*n* = 43), all treated laparoscopically. A number of patients required pouch revision (*n* = 9), often with replacement of the MiniMizer ring. Other frequent occurring complications consisted of food impaction (*n* = 4), diagnostic laparoscopic surgery without abnormalities (*n* = 7), and removal of the ring (*n* = 12).

There was no surgical-related mortality. Ten patients died during follow-up, but all deaths were attributed to causes unrelated to the surgical procedure. There was no statistically significant association between preoperative weight loss quartiles and the occurrence of overall postoperative complications (*p* = 0.474), short-term complications (*p 0.653)*, nor long-term complications (*p* = 0.660) (supplementary Table 1).

## Discussion

This study demonstrates that higher preoperative weight loss is associated with greater %TWL for the first post-operative period when stratified into four quartiles. Patients in the highest quartile (Q4) achieved significantly higher %TWL than those in lower quartiles till two years postoperatively. Although these differences were statistically significant, the absolute differences were small (2–3%). These modest differences suggest that while higher preoperative weight loss may offer a short-term advantage, its clinical impact is limited. By the third postoperative year, differences began to diminish and by five years, all groups converged to comparable %TWL across all quartiles.

When using a simpler (dichotomous) grouping, i.e. low versus high preoperative weight loss, the significant differences in %TWL continued until four years of follow-up. The overall pattern remained the same as early benefits were followed by convergence. These LMM findings were consistent with simpler year-by-year comparisons, confirming that differences between preoperative weight loss groups diminished over time. Continuous analysis confirmed this trajectory, showing a significant association between %preopWL and %TWL at one year but not at five years.

Several factors may explain why early differences vanish over time with all patients achieving a similar %TWL after five years. One explanation is the physiological mechanisms after MBS. For instance, a significant loss of lean body mass reduces resting energy expenditure which can increase hunger and energy intake over time [14]. This metabolic adaptation promotes weight regain and may offset early advantages. Additionally, hormonal and gut microbiome changes contribute to a new weight set point, further narrowing differences between groups. The analysis of postoperative weight loss excluding the preoperative component suggests a compensatory effect. Patients in the highest preoperative weight loss quartile lost less from surgery at 5 years compared to those in the lowest quartile. This indicates that greater preoperative weight loss does not translate into greater cumulative benefit. These findings underscore that mandated preoperative weight loss does not sustain long-term advantages in total weight loss. While early differences exist, they are modest and transient.

Our findings are comparable to the short-term results of previous research [9–11, 15]. For instance, one study found that patients who achieved 5–10% preoperative weight loss had significantly higher odds of achieving *≥* 25% TWL for two years follow-up compared to those with 0–5% weight loss [10]. Other studies also show an association between preoperative weight loss and greater total weight loss for the short-term follow-up [9, 11, 15]. However, some studies found no effect on postoperative outcomes. For example, Jacobs et al. reported minimal differences in postoperative TWL up to 3 years, even among patients with stable or increased preoperative weight [7]. Similarly, Eisenberg et al. reported that neither preoperative weight loss nor weight gain predicted postoperative BMI change at 1 year [16]. Patients who lost weight preoperatively had comparable BMI reduction compared to those who did not (34.6% vs. 34.5%, *p* = 0.79). There are several studies that report a short-term positive association, however others do not find significant differences in total weight loss over time [8, 17, 18]. These discrepancies may be due to variations in how weight loss is defined as some studies used %EWL [9, 15], others used %TWL [7, 10] and some combined metrics [11].

A potential explanation for the observed short-term benefit of preoperative weight loss may be due to behavioral and psychological factors such as motivation and adherence. Patients who succeed in losing weight before surgery may demonstrate higher levels of motivation, self-discipline, and adherence to lifestyle changes [3, 7]. However, these factors were not directly measured in this study and the ‘’motivation hypothesis’’ therefore remains speculative. Future research should incorporate validated assessments of diet adherence and social support.

Furthermore, this study also assessed the general predictors for total weight loss, independent of preoperative weight loss. Several baseline characteristics were associated with greater %TWL over time, up to five years. The positive predictors included female sex, younger age and RYGB procedure. These findings are consistent with general predictors already well established in MBS literature [19–22]. However, the effect of preoperative weight loss on %TWL was consistent across the quartiles. There were no significant interactions found with gender, age, BMI or procedure type. Although a trend suggested that males in the high weight loss group might benefit slightly more than females, this was not statistically significant.

There might also be another short-term advantage of preoperative weight loss as surgical circumstances are improved, among which reduction of liver volume [3, 8, 9]. It is expected that the reduced liver volume could shorten duration of the procedure and could lead to less blood loss and fewer complications. However, the study of Romaen et al. found no association between preoperative weight loss and (short-term) complication rates [3]. The long-term findings of this study are in accordance with the 1-year results of Romaen et al. [3]. There is no significant association between overall complications and preoperative weight loss. A possible explanation for this lack of association could be the relatively small number of severe short-term complications (1.6%) which is comparable to the national average of 1.2% CD3b complications reported in the Dutch Audit of Treatment for Obesity (DATO) [23]. The overall complication rate is low, and these small numbers may limit the statistical power to detect significant differences between groups.

### Strengths and Limitations

This study benefits from a large sample size, a standardized preoperative program, and extended five-year follow-up with high rates in the first two years. While previous studies have examined short-term associations few have assessed outcomes beyond three years. This study is one of the largest single-center cohorts (*n* = 765) with a controlled preoperative program to report 5-year outcomes. This minimizes variability and strengthens internal validity.

However, there are also some limitations. First, the absence of a true control group without preoperative weight loss limits the ability to determine whether any degree of preoperative weight loss is beneficial compared to none. As a result, even the ‘’lowest’’ quartile (Q1) still achieved measurable preoperative weight loss. Second, although one-year follow-up was high the rate declined to 65% at five years. This may introduce bias, although follow-up rates were similar across quartiles. It remains possible that patients that were loss to follow-up experienced different trajectories. If all patients lost to follow-up had poor outcomes, the overall %TWL would decrease, potentially weakening the conclusion of ‘no long term difference’ but most likely not reversing its direction. Finally, this was a single-center study conducted in a large teaching hospital. While this strengthens internal consistency, it may limit generalizability to other settings. Moreover, the cohort was predominantly treated with (ring augmented) RYGB, which is less common internationally. The SG subgroup was small, but its weight loss trajectory broadly mirrored the overall cohort. These findings remain relevant to centers where RYGB is the primary bariatric procedure, though extrapolation to centers favoring sleeve gastrectomy should be made with caution.

## Conclusion

Greater preoperative weight loss is associated with significantly greater %TWL during the early postoperative period, up to two years when analyzed by quartiles and up to four years when comparing high vs. low preoperative weight loss. These differences diminished over time, with no significant advantage at five years. The effect of preoperative weight loss on outcomes was consistent across subgroups and no patient group demonstrated a sustained benefit. Female sex, younger age, and undergoing RYGB were general predictors of greater %TWL, but these factors were independent of preoperative weight loss. Overall, mandated preoperative weight loss does not lead to a lasting benefit in total weight loss outcomes.

## Supplementary Information

Below is the link to the electronic supplementary material.

Supplementary Material 1

## Data Availability

No datasets were generated or analysed during the current study.
