# Supplementary material for: The Impact of Preoperative Weight Loss on Long-term Success: 5-year Outcomes After Metabolic Bariatric Surgery
Source: Obes Surg. 2026 Jan 6;36(2):738–46. doi: 10.1007/s11695-025-08476-2 (PMC12956915; doi:10.1007/s11695-025-08476-2)
Supplement: Supplementary file 1 — Supplementary Material 1 [file 11695_2025_8476_MOESM1_ESM.docx]

|  | Q1  (n=191) | Q2  (n=187) | Q3  (n=196) | Q4  (n=191) | *p-value* |
| --- | --- | --- | --- | --- | --- |
| Age in years | 43 (31 – 51) | 44 (33 – 51) | 47 (37 – 53) | 46 (36 – 54) | **0.011** |
| Gender  *Female*  *Male* | 149 (78)  42 (22) | 150 (80.2)  37 (19.8) | 151 (77)  45 (23) | 136 (71.2)  55 (28.8) | 0.195 |
| Max BMI | 42.4 (40.1 – 47.5) | 42 (39.4 – 45.1) | 41.5 (39.1 – 44.6) | 42.2 (40 – 46.3) | 0.063 |
| Type of surgery  *RYGB*  *Sleeve* | 169 (88.5)  22 (11.5) | 168 (89.8)  19 (10.2) | 181 (92.3)  15 (7.7) | 179 (93.7)  12 (6.3) | 0.264 |
| Total complications | 22 (11.5) | 24 (12.8) | 32 (16.3) | 30 (15.7) | 0.474 |
| Clavien-Dindo >3B | 21 (11) | 17 (9.1) | 25 (12.8) | 23 (12) | 0.191 |
| Short-term complications | 3 (1.6) | 6 (3.2) | 7 (3.6) | 6 (3.1) | 0.653 |
| Long-term complications | 19 (9.9) | 18 (9.6) | 25 (12.8) | 24 (12.6) | 0.660 |
| Missing data at 5 year follow-up | 62 (32.5) | 65 (34.8) | 68 (34.3) | 77 (40.3) |  |

**Supplementary table 1. Baseline characteristics per quartile**

*Data presented as median (IQR) or N (%). P-value calculated with Chi-square for categorical variables and Kruskal-Wallis for continuous variables*

**Supplementary table 2. Adjusted predictors of %TWL at 1 and 5 years after MBS**

| **Predictor** | **1-year β** | **p-value** | **5-year β** | **p-value** |
| --- | --- | --- | --- | --- |
| **Pre-op %TWL** | 0.45 | <0.001 | -0.46 | 0.415 |
| **Age (per year)** | -0.14 | <0.001 | -0.41 | 0.012 |
| **Gender (female)** | 2.55 | <0.001 | 6.03 | 0.176 |
| **Sleeve vs RYGB** | -2.37 | 0.004 | -2.46 | 0.701 |
| **Baseline BMI** | 0.06 | 0.170 | 0.43 | 0.216 |

*P-value calculated with multivariable linear regression*
